# Oral findings in chronic kidney disease: implications for management in developing countries

**DOI:** 10.1186/s12903-015-0004-z

**Published:** 2015-02-20

**Authors:** Elijah O Oyetola, Foluso J Owotade, Gbemisola A Agbelusi, Olawumi A Fatusi, Abubarkar A Sanusi

**Affiliations:** Department of Preventive and Community Dentistry, Obafemi Awolowo University Teaching Hospitals Complex, Ile Ife, Osun State Nigeria; Department of Oral and Maxillofacial Surgery and Oral Pathology, Obafemi Awolowo University Teaching Hospitals Complex, Ile Ife, Osun State Nigeria; Department of Preventive Dentistry, Lagos University Teaching Hospital, Idi- Araba, Lagos State Nigeria; Department of Internal Medicine, Obafemi Awolowo University Teaching Hospitals Complex, Ile Ife, Osun State Nigeria

**Keywords:** Oral lesions, Chronic kidney disease, Abnormal lip hyperpigmentation, Gum bleeding

## Abstract

**Background:**

The importance of oral health care in the management of patients with systemic diseases including chronic kidney disease (CKD) has been affirmed. Many CKD patients have related oral lesions, however, attention to oral health care has been lacking, especially in the developing countries with higher burden of renal diseases.

**Methods:**

One hundred and eighty patients, 90 cases and 90 controls were recruited, interviewed and examined. Oral mucosa assessment was based on the WHO Guide to Epidemiology and Diagnosis of Oral Mucosal Diseases. Urinalysis and blood creatinine levels were determined. Glomerular filtration rate (GFR) of each patient was calculated from the blood creatinine using Cockcroft and Gault formula.

**Results:**

Oral lesions were present in 86 out of 90 (96.5%) CKD patients compared with 15 out of 90 (16.7%) controls (p < 0.001). Abnormal lip hyperpigmentation was the most frequently seen lesion in 81 out of 90 (90%) CKD patients. Other significant findings were gum bleeding, xerostomia, candidiasis, burning mouth and abnormal taste. In the controls (without CKD), the mean GFR was lower in subjects with oral lesions compared with those without oral lesions p < 0.001.

**Conclusions:**

CKD and reduced GFR in subjects without CKD are risk factors for oral lesions. The higher prevalence of oral lesions in CKD patients necessitates mandatory oral screening to identify patients with deteriorating renal function. The management of such lesions will enhance the overall well-being of CKD patients in developing countries.

## Background

The mouth is a powerful diagnostic tool in the clinical assessment of systemic health [1]. Peculiar oral findings have been reported in diseases such as Human Immunodeficiency Virus (HIV) infection where unusual oral lesions have aided the diagnosis of HIV infection among routine dental patients [2]. Other diseases where oral manifestations may play a vital role in the diagnosis and management include diabetes mellitus [3], coronary heart disease [4], graft versus host disease [5] and chronic kidney disease [6].

Generally, the reported oral lesions in systemic illness include periodontitis, white patches, red patches, mucositis, oral candidiasis, burning sensation, changes in salivary composition and flow rates, pale mucosa and abnormal pigmentation [6,7]. The presence of these specific oral lesions is not only helpful in detecting underlying systemic diseases but may also indicate the severity of such systemic diseases [8]. In addition, significant improvement in systemic health has been reported following the treatment of associated oral lesions [6,8].

Chronic kidney disease (CKD) like many other systemic diseases, have associated oral problems arising from the disease process or the effects of therapy or both. Consequently, untreated oral lesions may worsen the clinical presentation and prognosis [8]. The National Kidney Foundation (NKF) defined chronic kidney disease as kidney damage for three or more months associated with structural or functional abnormalities of the kidney, with or without decreased glomerular filtration rate (GFR) [9]. Furthermore, CKD is a public health problem with a high impact on quality of life [10]. CKD incidence is 337 per million in United States of America and 95 per million populations in United Kingdom [11]. In Nigeria, the incidence is 1.8-10% (1,800-10,000 per million population) and it represents 27.17% of all medical outpatients’ clinic attendance. CKD is therefore regarded as one of the major public health problems in Nigeria [12].

CKD is associated with clinical and radiographic changes in the mouth [6,11]. The radiographic changes include loss of lamina dura, maxillary and mandibular radiolucent lesions [11]. The clinical findings in CKD patients are essentially as stated for oral lesions in systemic disease [6]. Unfortunately, attention to the oral aspects has been lacking despite the many merits associated 13]. High graft rejection rate (in kidney transplant patients) [13] and increase in systemic inflammatory burden which worsen the underlying systemic disease [8] are consequences of untreated oral lesions in CKD.

The cause effect relationship between oral infections and systemic diseases is yet to be fully established; however significant improvement in the underlying systemic diseases has been reported following treatment of associated oral lesions [14]. This is a pointer to the importance of oral health care in the management of systemic diseases. In the developed countries, there are guidelines for oral health care in patients with systemic diseases [15]. The reverse is the case in the developing countries where inadequate attention has been given to oral health care needs in this group of patients and this has further worsened the prognosis of the underlying disease [13]. Poor dental awareness among the patients and some medical colleagues are contributory factors [16].

This study investigated oral lesions in CKD in our environment and the association with renal function impairment. An oral health care protocol for the management of CKD patients was also suggested.

## Methods

### Study design

The study was a case control study comparing oral lesions in CKD patients attending the renal clinic of the Obafemi Awolowo University Teaching Hospitals Complex, Ile Ife, Nigeria with controls from the General Medical Outpatient Clinic between September 2011 and March 2012.

### Subjects

The study group consisted of patients diagnosed with chronic renal failure and end stage renal disease. This group was randomly selected from the pool of CKD patients being managed by the Renal Unit of the Obafemi Awolowo University Teaching Hospital Complex, Ile-Ife, Nigeria. Subjects selected were 18 years and above with no co-morbid medical conditions like diabetes mellitus and primary hypertension as well as negative history of cigarette and alcohol consumption.

The control group comprised clinically healthy subjects who visited the general out-patient department (GOPD) of the hospital on account of pre-employment, pre-admission and routine medical checkup. Control subjects were recruited if they had normal estimated glomerular filtration rate (eGFR) (GFR above 120 ml/min per 1.73 m^2^ surface area). The GFR was estimated using the Cockcroft and Gault equation [17]. Other inclusion criteria for control subjects were negative history of kidney disease or other chronic debilitating illnesses, cigarette smoking or alcohol consumption.

### Ethical issues

Ethical clearance was obtained from the Ethics and Research committee (ERC), Obafemi Awolowo University Teaching Hospitals Complex, Ile-Ife.

### Sample size estimation

Xerostomia, previously estimated to be 20% prevalent in the adult population was used to estimate the sample size [18]. With alpha set at 5%, power at 90% and prevalence of xerostomia projected to be 45% in renal patients as reported by De la Rosa et al. [19], 80 subjects were required in each group.

### Data collection

Patients who met the inclusion criteria were informed about the study after which a signed consent was obtained. Information on patients’ biodata, relevant medical history, drug history, alcohol and tobacco consumption was recorded in addition to the blood pressure and weight of subjects.

Detailed extra oral and intraoral examination was done, and organoleptic method was used to assess halitosis. Oral mucosa assessment was based on the WHO Guide to Epidemiology and Diagnosis of Oral Mucosal Diseases [20].

#### Periodontal health assessment

Oral hygiene was assessed using Greene and Vermilion oral hygiene index [21]. Generally, a tooth is said to have developed periodontitis when there is an established pocket of more than 3 mm depth [22]. For this study, teeth with established pocket more than 3 mm were described as positive for periodontitis. Periodontal pocket was measured with the Magil ‘O’ periodontal probe which has marking at 3 mm point from the tip and thus making it easier to pick subjects with periodontics (periodontitis in this study is set at a pocket of 3 mm and above), it is also a readily available periodontal probe [23].

#### Gingival status

Gingival status was assessed using gingival index of Loe H (1967) [24].

#### Salivary measurements

Objective salivary measurements (unstimulated whole saliva (UWS) and stimulated whole saliva (SWS) was done using spitting method [25].

All the examination/measurements were carried out by one author (Oral Medicine Specialist) after prior calibration. The first twenty patients were examined by two senior specialists. The inter examiner consistency was determined with Kappa statistics. The Kappa coefficient for this study was 0.91 which was found to be appropriate [26].

### Assessment of renal function

Urinalysis was done for each patient using Combic 9® and blood creatinine was determined from which the estimated GFR of each subject was calculated using Cockcroft and Gault equation [17], as follows:

$$ \begin{array}{l}\mathrm{Estimated}\ \mathrm{creatinine}\ \mathrm{clearance}\ \left(\mathrm{ml}/ min\right) = \left(140\hbox{-} \mathrm{age}\right)\ \mathrm{X}\ \mathrm{body}\ \mathrm{weight}\\ {}\kern17.25em 72\ \mathrm{X}\ {\mathrm{P}}_{\mathrm{cr}}\left(\ \mathrm{mg}/\mathrm{dl}\right)\end{array} $$

For female subjects, the gender correlating factor was applied [17].

### Statistical analysis

Descriptive statistics, bivariate analysis such as *t*-test, Fisher’s exact and chi-square statistics or their non-parametric equivalents were used as appropriate to compare the two groups. Multivariable logistic regression was used to determine the predictors of oral lesions. Data analysis was done using Stata 11 statistical software (Statacorp, College Station, Texas) and statistical significance was inferred at p < 0.05.

## Results

### Distribution of the subjects

One hundred and eighty subjects participated in the study, 108 (60%) males and 72 (40%) females. Average age of the subjects was 47.73 ± 16.08 years and majorities (96.7%) of the subjects were of Yoruba ethnicity (Table 1).

**Table 1:** **Sociodemographic factors and oral complaints**

**Characteristics**	**All**	**CKD**	**Controls**	**p value**
	**n = 180**	**n = 90**	**n = 90**	
**Average age (SD)**	47.73 (16.1)	50.39 (15.3)	45.18 (16.5)	
**Sex**				
Male	108 (60)	63 (58)	45 (42)	
Female	72 (40	27 (37.5)	45 (62.5)	<0.001*
**Ethnicity**				
Yoruba		87 (96.7)	82 (91.1)	
Hausa		1 (1.1)	1 (1.1)	
Ibo		7 (7.8)	2 (2.2)	0.064
**Oral lesions** [n (%)]				
Present	102 (56.7)	87 (96.7)	15 (16.7)	
Absent	78 (43.3	3 (3.3)	75 (83.3)	<0.001*
**Burning mouth** [n (%)]	16 (8.9)			
Present		16 (18)	0 (0)	<0.001*
Absent		74 (82)	90 (100)	
**Abnormal taste** [n (%)]	24 (13.3)			
Present		23 (26)	1 (1)	<0.001*
Absent		67 (74)	89 (99)	
**Bleeding gums** [n (%)]	22 (12)			
Present		21 (23)	1 (1)	
Absent		69 (77)	89 (99)	<0.001*
**Halitosis**	12 (7)			
Present		11(12)	1 (1)	
Absent		79(88)	89 (99)	0.005
**Xerostomia**	13 (7.2)			
Present		11 (2)	2(2)	
Absent		79 (88)	88 (98)	0.018

Fisher’s exact, *statistically significant.

#### Oral soft tissue lesion

Abnormal lip pigmentation was the most frequent lesion seen in CKD subjects, seen 81 out of 90 CKD subjects (90%). Other significant lesions seen include candidiasis, pale mucosa, petechial hemorrhage and periodontitis (Table 2).

**Table 2:** **Oral soft tissue lesions**

**Characteristics**	**All**	**Cases**	**Controls**	**p value**
	**n = 180**	**n = 90**	**n = 90,50%**	
Abnormal lip pigmentation [n (%)]				
Present	86 (48)	81 (90)	5 (6)	<0.001*
Absent		9 (10)	85 (94%)	
Oral *Candida* infection [n (%)]	15 (8.3)			
Present		14 (15.5)	1 (1)	<0.001*
Absent		76 (84.5)	89 (99)	
Macroglossia [n (%)]	2 (1)			
Present		2 (2)	0 (0)	
Absent		88 (98)	90 (100)	0.497
Depapillated tongue [n (%)]	3 (1.7)			
Present		3 (3.3)	1 (1)	
Absent		87 (96.6)	89 (99)	0.246
Petechial haemorrhage [n (%)]	10 (5.6)			
Present		10 (11.1)	0 (0)	<0.001*
Absent		80 (89.9)	90 (100)	
Hyperpigmented mucosa	12 (7)			
Present		11 (12)	1 (1)	
Absent		79 (88)	89 (99)	0.005*
Aphthous ulcers [n (%)]	2 (1.11)			
Present		2 (2)	0 (0)	
Absent		78 (88)	90 (100)	0.247
Pale mucosa [n (%)]	22 (12)			
Present		22 (24)	0 (0)	
Absent		68 (76)	90 (100)	0.004*
Hyperemic mucosa [n (%)]	13 (7.2)			
Present		13 (14.4)	0 (0)	
Absent		77 (85.6)	90 (100)	<0.001*
Gingivitis [n (%)]	175 (97.2%)			
Present		88 (97.7)	86 (95.5)	
Absent		2 (2.3)	4 (3.5)	1.000
Periodontitis [n (%)]	57 (31.2)			
Present		47 (52)	10 (11.1)	
Absent		43 (48)	80 (88.1)	<0.01*
Gingivae recession [n (%)]	39 (21.6)			
Present		35 (38.9)	4 (4.4)	
Absent		55 (61.1)	86 (95.6)	<0.01

Fisher’s exact, *statistically significant.

#### Glomerular filtration rate and presence of oral lesions

A positive relationship was found between the estimated GFR and oral lesions in cases and in controls, and this was statistically significant as shown in the Table 3.

**Table 3:** **Glomerular filtration rate and presence of oral lesions**

	**GFR of Subjects with oral lesions (ml/min)**	**GFR of subjects without oral lesions (ml/min)**	**P.value**
CKD subjects	49.7 ± 22.4	77.7 ± 45.3	<0.01*
Control	109.5 ± 20.6	113.5 ± 17.7	<0.01*
All subjects (Cases and control combined)	58.5 ± 30.6	112.2 ± 20.1	<0.01*

*t- test with unequal variances.

#### Mean GFR and oral lesions in CKD subjects

In this study, aphthous ulcers were seen in CKD subjects with high mean GFR(57.6 ml/min per 1.73 m^2^ surface area) unlike oral candidiasis which was seen in subjects with low mean GFR( 35.5 ml/min per 1.73 m^2^ surface area). Other lesions seen at a significantly lower mean GFR are: xerostomia, halitosis and abnormal lip pigmentation seen in subjects with mean GFR of 38.6 ml/min per 1.73 m^2^ surface area, 39.7 ml/min per 1.73 m^2^ surface area and 49.7 ml/min per 1.73 m^2^ surface area respectively.

#### Relationship between oral lesions and blood urea concentration in renal patients

The mean blood urea concentration in CKD patients with oral lesions (9.4 mmol/L) was higher compared with those without oral lesions (5.1 mmol/L) although the difference was not statistically significant P = 0.08. Blood urea nitrogen was highest in patients with oral candidiasis (12.5 mm/L) followed by those with halitosis (12.1 mmol/L), xerostomia (11.6 mmol/L), pale mucosa (11.1 mmol/L) and periodontitis (9.4 mmol/L).

### Regression analysis

Table 4 shows the association between chronic kidney disease and development of oral lesions after adjusting for age and sex. The likelihood of developing oral lesions was significantly higher in subjects with CKD compared with controls (Odds ratio 153.3, 95% C.I (40.1-584.8), p < 0.001). Age and sex did not have a significant relationship with the likelihood of developing oral lesion.

**Table 4:** **Role of chronic kidney disease on development of oral lesions**

**Covariate**	**Odds ratio**	**Standard error**	**95% CI**	**p value**
CKD	153.5	104.3	40.5-581.4	<0.001*
Age	1.03	0.17	1.0-1.06	0.45
Sex (Male)	0.93	0.33-2.6	0.33-2.63	0.89

*Statistically significant, Hosmer-Lemeshow goodness of fit test p = 0.1914.

## Discussion

Oral lesions were present in 87 of 90 subjects with CKD, representing a prevalence of 97%. This is consistent with studies from different parts of the world excluding Africa that reported up to 90% of CKD patients showing oral symptoms in the course of their kidney disease [11,27]. In some reports, prevalence of oral lesions in CKD was 100% [6]. Oral lesions are usually due to restricted diets, malnutrition, mouth neglect, immunosuppression and the effects of medications and ureamic toxins on the oral tissues [11]. CKD patients on hemodialysis have also been found to be associated with reduced dental visits which further worsen the oral care [28]. The high prevalence in this study may be attributed to the peculiarity of patients’ presentation and local factors that affect the choice of the treatment options affordable in this part of the world. Most Nigerians present late to health facilities and oftentimes would have tried various forms of alternative medicine like spiritual and traditional/native healers before presenting to a health facility [12,29]. This late presentation is due to ignorance and poor accessibility and affordability of health care services [30]. Majority of patients, therefore, present at late stages when oral lesions were more likely prevalent. Oral lesions were more prevalent in males (64%) and this is consistent with other reports who attributed this to the predilection of CKD for males [11]. Oral lesions (pathologic conditions in the mouth), oral signs (abnormalities of oral mucosa found on clinical oral examinations) and oral complaints (described by patients) are the various terms used in describing the oral aspects of kidney disease in the literature [31].

Abnormal lip pigmentation, the most prevalent oral lesion observed in our CKD patients was not commonly reported among renal patients in the earlier studies [11,27]. Oral and cutaneous hyperpigmentation in renal patients is due to inability of the kidney to excrete excess beta melanocyte stimulating hormone (b-MSH), the accumulation of which results in the stimulation of melanocyte at the basal layer of oral epithelium [32]. The possible reasons for the high frequency of hyperpigmentation in our environment may be due to the abuse of traditional or orthodox drugs for the treatment of kidney disease or other diseases. These drugs which include antimalarials (quinacrine, chloroquine, hydroxychloroquine) may stimulate melanin secretion and are easily accessible in our environment [33]. In addition, these drugs which are also known to induce hypermelanosis are frequently used by these patients before presentation. Genetic factors (black race) and hot climate associated with constant exposure of the melanocytes to sunlight may also hasten hyperpigmentation. Consequently, abnormal lip and face pigmentation constitutes a great aesthetic challenge to renal patients [34].

Abnormal taste has been commonly reported and was present in 23 (26%) of the CKD subjects [11,35]. The mechanisms underlying alterations in taste perception in uremia patients are unknown, but are probably attributable to influences of ureamic toxins on the central nervous system (CNS) and the peripheral nervous system (the taste receptors) [36]. Bleeding gum was seen in 23 out of 90 (26%) CKD subjects and has been attributed to poor oral hygiene, ginigival/periodontal inflammation and bleeding abnormalities [37]. Similarly, burning mouth sensation which was significantly higher in CKD patients (16%) was a consistent finding in many other studies. It was attributed to effects of dry mouth, damage to peripheral nerves by the ureamic toxins and effects of medication [11,38].

In CKD, there is usually fluid restriction, electrolyte imbalance, and use of medications, such as frusemide and hydrochlorothiazide. These may contribute to the complaint of a dry mouth seen in 12.22% of the renal patients in our study. Other researchers documented a much higher prevalence of 48.2% [39] and 32.9% [40]. The higher values in studies conducted outside sub Saharan Africa may be due to the primary aetiology of kidney disease in these regions. The aetiological factors in these regions such as diabetes mellitus, hypertension, amyloidosis and autoimmune disease not only cause renal disease but also initiate salivary gland disease independently [12]. In Africans, the commonest aetiology of renal disease is glomerulonephritis which usually results from infections [29]. The complaint of halitosis was significantly higher in CKD patients (12%) and has been attributed to dry mouth, poor oral hygiene and ureamic smell [11].

The lower mean stimulated and unstimulated salivary production found in CKD patients (2.34 ml/5 min and 4.07 ml/5 min) when compared to the controls (3.82 ml/5 min and 8.05 ml/5 min) was consistent with majority of earlier studies [11,41,42]. This finding is similar to that of Kho and colleagues [40] who reported 0.44 + 0.29 ml/min and 1.5 + 0.5 ml/min for unstipulated whole salivary (UWS) flow rate in renal patients and controls respectively. Reduced salivary flow rate is a consistent finding in CKD patients and it has been reported that reduce flow is due to effects of drugs, emotional stress and neuropathy in CKD patients [43].

Oral candidiasis was more prevalent in CKD cases and may be due to immune suppression from malnutrition, restricted diets, anaemia, stress, and immunosuppressive drugs. Result of this study was consistent with the findings of Al-Mohaya [44] and colleagues who reported 15.5%, and De la Rosa-Garcia [45] (18.8%). However these studies were carried out among post-renal transplant chronic kidney disease patients. Considering a higher prevalence of oral candidiasis (37%) reported by Royne and colleagues [46] and lower prevalence of 5.7% reported by Gavalda and co-workers [47], oral candidiasis appears to be a consistent finding in patients with chronic kidney disease [11,27]. Late presentation of patients, environmental factors and possibly genetic variations may account for the little variation between our study and the above mentioned studies.

Gingival swelling, described by Proctor and colleagues[11] and Al-Mohaya and co-workers [44] as the commonest oral manifestation of renal disease was not observed in this study despite the prevalent poor oral hygiene. Gum swellings arise from use of medications such as nifedipine, cyclosporine and tacrolimus [6,11,44]. These drugs are not routinely used in the management of renal cases in our hospital. The drugs used in our hospital are lisinopril and amlodipine for the treatment of hypertension in renal patients with other supportive drugs. These drugs (lisinopril and amlodipine), unlike nifedipine and cyclosporine, are not associated with gum swellings. Furthermore, most of our patients cannot afford the cost of renal transplantation, a peculiar problem in this environment and developing nations [29]. Such patients (which constitute the majority) are placed on palliative management consisting of the pharmacodietary approach, without the need for immune suppressant drugs that may predispose to gingival swelling.

### Glomerular filtration rate and oral findings

Majority of the studies reported a GFR range of 90-120 ml/1.73 m^2^ surface area as normal for an adult, with lower values indicating poor renal function [6,17,37]. It was observed that the mean GFR of subjects with oral lesions was significantly lower than mean GFR of those without oral lesion p < 0.001, showing a strong relationship between presence of oral lesions and reduction in GFR especially in CKD subjects. Systemic inflammation arising from oral infection could have an effect on microvasculature of heart and kidney [4,6]. The possible establishment of bi-directional relationship between oral infection such as between periodontitis and heart disease has also been postulated between oral lesion and kidney disease [6,8]. This probably explains the lower mean GFR in CKD subjects with oral lesions when compared with a relatively higher mean GFR in CKD subjects without oral lesions.

## Conclusion

The prevalence of oral lesions was higher in CKD subjects than in controls and the difference was statistically significant. Abnormal lip pigmentation, halitosis, periodontitis and candidiasis are some of the oral lesions seen. The presence of oral lesions was also associated with reduced GFR in both CKD and control subjects. The positive association between the presence of oral lesions and reduced GFR in healthy/control subjects is a pointer to the possible role(s) of oral lesions in the initiation and/or progression of renal disease.

**Figure 1:**
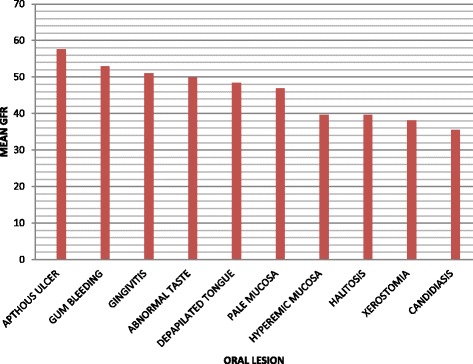
**Mean GFR and oral lesions in CKD subjects.**

In addition, this study showed the possibility of using presence of oral lesions to predict the severity of underlying renal problem as shown in Figure 1 where the relationship between the mean GFR and oral lesions were clearly shown. While the presence of oral candidiasis (seen in subjects very low GFR) may indicate a state of severe renal impairments, the presence of lesions like aphthous ulcer may reflect a just mild to moderate renal disease since they are usually seen at slightly below normal GFR.

Sequel to the impact of oral aspect of CKD in the quality of life of patients, it is therefore recommended that all patients with chronic kidney disease will benefit from dental care; therefore, they should be routinely evaluated for oral lesion(s) and treated accordingly. Likewise, dental evaluation and appropriate dental treatments should be given to all prospective kidney transplant patients before transplantation as well as regular review after the transplantation. More importantly, there is need for a closer relationship between nephrologists and dentists in the management of chronic renal patients so as to ensure optimal quality of life at all times.

## Abbreviations

CKD: Chronic Kidney Disease
GFR: Glomerular filtration rate
HIV: Human Immunodeficiency Virus
NKF: National Kidney Foundation
